# A Novel Temporary Anchorage Device Aided Sectional Mechanics for Simultaneous Orthodontic Retraction and Intrusion

**DOI:** 10.1155/2020/5213936

**Published:** 2020-01-29

**Authors:** Adith Venugopal, Paolo Manzano, Sri Rengalakshmi

**Affiliations:** ^1^Section of Orthodontics, University of Puthisastra, Phnom Penh, Cambodia; ^2^10:10 Dental Clinic, Mandaluyong, Manila, Philippines; ^3^Department of Orthodontics, Saveetha Dental College, Tamil Nadu, India

## Abstract

Closing spaces on light wires with inadequate knowledge and inappropriate mechanics can cause a “roller coaster” effect leading to an improper occlusion. Current knowledge of biomechanics, along with the incorporation of TADs, has made this process less challenging and more predictable. Resistance to sliding is considered the most prominent inhibitor of space closure in archwire-guided space closure or sliding mechanics, in turn delaying treatment duration considerably. In our case, resistance to sliding, primarily binding of the wire in the bracket slot, was nullified with the use of loop and sectional mechanics. This case report is aimed at showcasing the successful treatment of a young lady with a novel clinical setup to retract the canines into the premolar extraction space and simultaneously retract and intrude the anterior segment using sectional archwires and TADs in just under nine months. The current setup with sectional wires and TADs produced an uprighting, and an intrusive effect on the upper incisors during space closure. Additionally, the anchorage design avoided any significant change in the vertical dimension during sagittal correction of the Class II malocclusion. The occlusal plane remained almost stable with good amount of uprighting of the lower incisors following lower space closure too. The use of good biomechanical principles helped us achieve all the treatment goals and objectives in a very short period of time.

## 1. Introduction

Closing edentulous sites in orthodontics is a challenging task. Using light wires with inadequate knowledge and inappropriate mechanics in this task can cause a “roller coaster” effect leading to an improper occlusion [1]. Current knowledge of biomechanics, along with the incorporation of temporary anchorage devices (TADs), has made this process less challenging and more predictable [1–4].

“Friction” has received much attention in orthodontics, especially when it comes to space closure. Resistance to sliding is considered the most prominent inhibitor of space closure in archwire-guided space closure or sliding mechanics. Resistance to sliding is caused due to three components. These are (1) classical friction, (2) binding, and (3) notching. Binding and notching have been proved to play an essential role in the resistance to sliding [5]. It is clear from the current literature that resistance to sliding is an unavoidable concern in archwire-guided tooth movement or sliding mechanics, especially in closing extraction spaces and in turn prolongs the treatment duration considerably [6].

Segmented arch mechanics was proposed to have better-controlled tooth movement than archwire-guided tooth movement and is multifactorial, as reflected in the literature. Segmented arch mechanics suggests dividing the dental arch into three major segments (especially in extraction cases) [3]. These three segments are one anterior (incisors and canines) and two posteriors (teeth posterior to the extraction site). The canines are retracted first into the extraction spaces, followed by retraction of the incisors. This technique is aimed at minimizing posterior teeth movement forward, also known as maximum anchorage [7, 8]. Unfortunately, this technique also prolongs treatment duration due to its two-step nature.

This case report is aimed at showcasing the successful management of the spaces required to improve the esthetics and function of an adult female through the simultaneous retraction and intrusion of the whole anterior segment. This technique was achieved through the retraction of the canines with the posterior teeth and TADs as anchorage, while the four incisors were retracted and intruded using the TADs combined with sectional archwires in just under nine months.

## 2. Diagnosis and Treatment Planning

**Figure 1:**
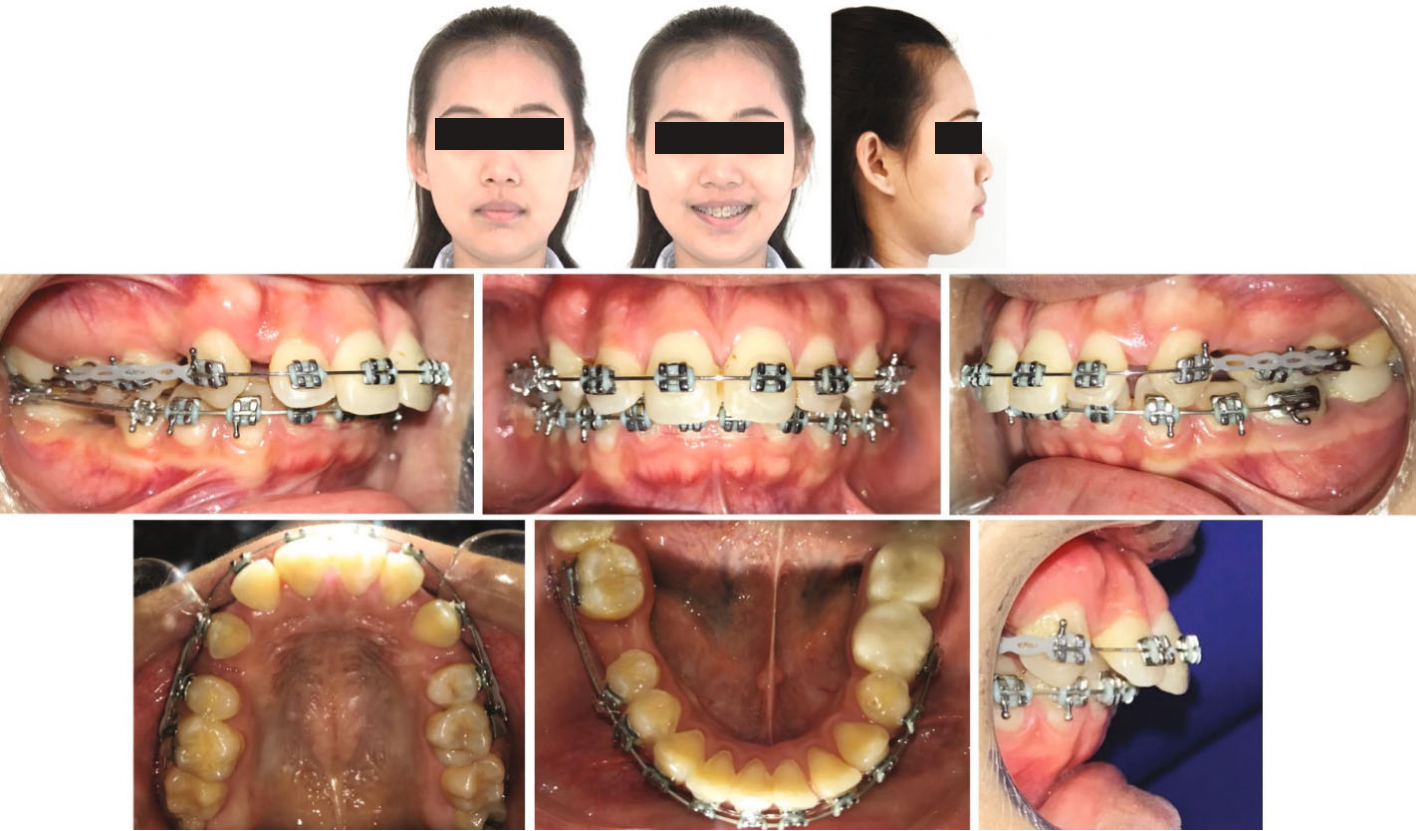
Pretreatment records with prior braces. 0.22^″^ MBT prescription brackets seen with canines half retracted on a 0.016^″^ NiTi archwire.

A 25-year-old female patient presented to our office with prior braces. On clinical examination, she presented with a convex profile, vertical growth pattern, slight gummy smile, extracted teeth 1.4 and 2.4, noncoincident midlines, increased overjet of 8 mm, and a deep bite. Canines were retracted halfway through on a 0.016^″^ nickel-titanium (NiTi) wire on 0.022^″^ slot MBT prescription brackets (Figure 1).

**Figure 2:**
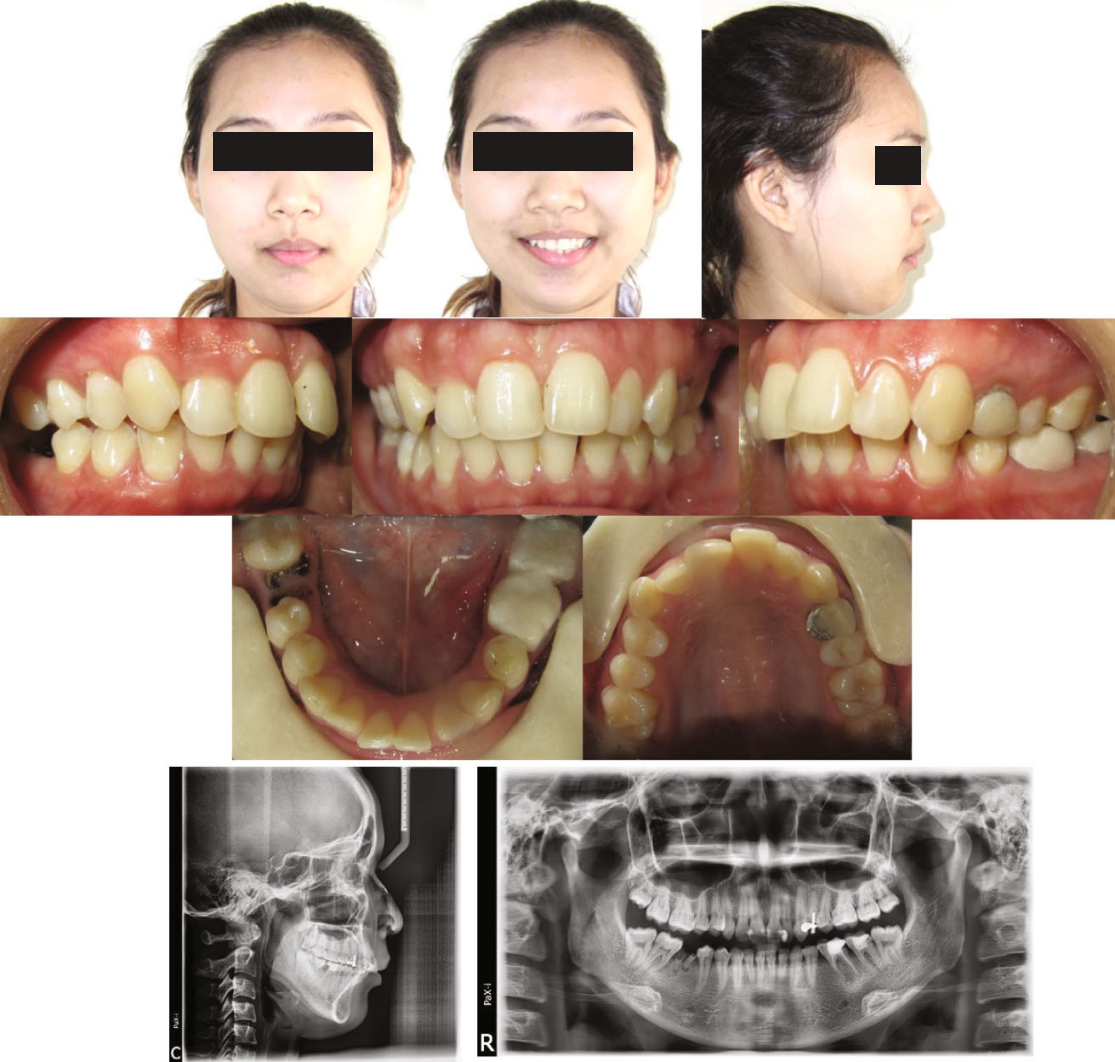
Initial treatment records prior to bonding with her previous orthodontist from two years ago.

The patient had brought along her pretreatment records along for evaluation. Initial pretreatment extraoral records revealed a convex profile with incompetent lips. Pretreatment intraoral records revealed an overjet of 5 mm, visible buccal corridors, and narrow maxillary arch with Class II canines on the left and the right. Root stumps in relation to 4.6 and spaces between her lower anterior teeth were present (Figure 2).

Radiographic evaluation of the initial pretreatment records revealed a slightly retrognathic mandible (SNB, 78.88°), steep mandibular plane angle (FMA, 28.94°) with proclined upper and lower incisors (U1-SN, 109.45°; IMPA, 101.07°). Tooth numbers 2.4 and 3.6 had undergone prior endodontic treatment (Figure 2 and Table 1).

### 2.1. Treatment Objectives

The patient had been undergoing orthodontic treatment for two years already before visiting our practice. The patient expressed psychological frustration with the existing treatment due to its duration and the mounting pressure for her need to leave the country in the next nine months. She stressed it enough that she was not willing to continue her orthodontic treatment elsewhere and would have the appliance removed, whether completed or not.

With the patient's wishes in mind, we decided to fabricate realistic goals and objectives, which were to correct the severe deep bite, close the residual spaces on the upper and lower arches to achieve ideal overjet and overbite, achieve Class I canine relationships, and achieve a pleasant profile. One of the objectives was also to maintain space for an extra premolar prosthesis in the 4^th^ quadrant as there was insufficient time to close the molar space.

### 2.2. Treatment Alternatives

Three alternatives were constructed in order to achieve the treatment objectives successfully.

- The first option would be to bond the second molars and relevel and align the arches followed by using an intrusion arch to correct the deep bite. Then, the canine retraction could be continued on a heavy stainless steel archwire followed by anterior retraction using loop or sliding mechanics by using the posterior segment as anchorage
- The second option was to bond the second molars and use an intrusion arch to correct the deep bite followed by protraction of the canines. Then, two TADs could be placed between the upper second premolars and first molars (1.5–1.6, 2.5–2.6) followed by en masse retraction by sliding mechanics
- The third option was to design a mechanotherapy which would allow simultaneous canine retraction using a section of wire with a closing loop along with simultaneous anterior intrusion and retraction using sectional wires and three TADs. Two of which would be placed between the upper first and second molars (1.6–1.7, 2.6–2.7) and the third between the two upper central incisors (1.1–2.1)

After careful consideration of all the three treatment options, the third option was finalized due to the given time constraints and was thoroughly explained to the patient.

### 2.3. Treatment Progress

#### 2.3.1. Canine Retraction, Anterior Retraction, and Simultaneous Intrusion

The design consisted of three segments of wires. One segment of 0.019^″^ × 0.025^″^ SS wire extended from tooth 1.2 to 2.2. Hooks were extended on the wire distal to the lateral incisor brackets to reach close to the center of resistance of the anterior segment.

**Figure 3:**
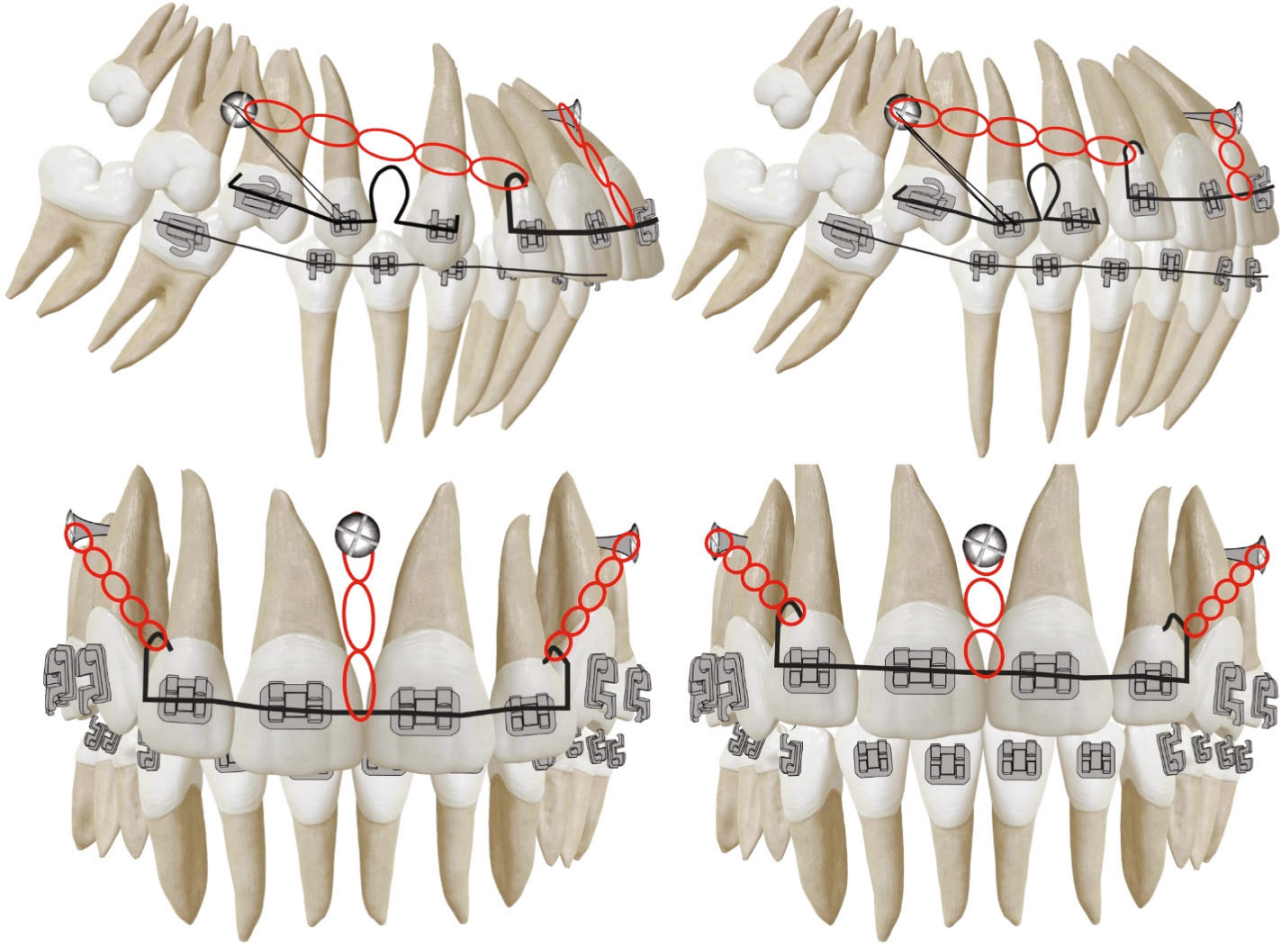
Biomechanical setup for canine retraction and simultaneous anterior retraction and intrusion. TADs placed between upper second premolar and first molars and also between upper central incisors. Sectioned 0.019^″^ × 0.025^″^ SS archwires anteriorly and posteriorly.

Posterior segments consisted of a segment of 0.019^″^ × 0.025^″^ SS extending from the first molar to the canines on either side. A closing loop was fabricated on the segment just distal of the canines (Figure 3).

Three TADs (1.6 × 8 mm) were inserted, two of them were placed between the upper first and second molars (1.6-1.7, 2.6–2.7) and the third between the two upper central incisors (1.1–2.1) (Figure 3).

#### 2.3.2. Activation

Elastic traction using a power chain from the posterior TADs was directed to the hooks fabricated on the anterior segment of wire just distal to the lateral incisor brackets in order to retract the anterior segment. The force was measured to be 150 grams per side using a Dontrix force gauge. Elastic traction using an elastic thread was applied from the anterior miniscrew to the sectional archwire between the two incisors in order to prevent the uncontrolled tipping, which would be a direct result of the posterior traction. At the same time, this would help intrude the anterior segment eventually correcting the deep bite. The force for intrusion was measured to be 60 grams.

The posterior segment was activated by cinching the wire posteriorly distal to the first molar and anteriorly mesial to the canines, thereby activating the closing loop, eventually retracting the canines. A passive ligature was tied from the posterior TADs to the second premolar brackets for indirect anchorage in order to prevent molar mesialization during canine retraction.

**Figure 4:**
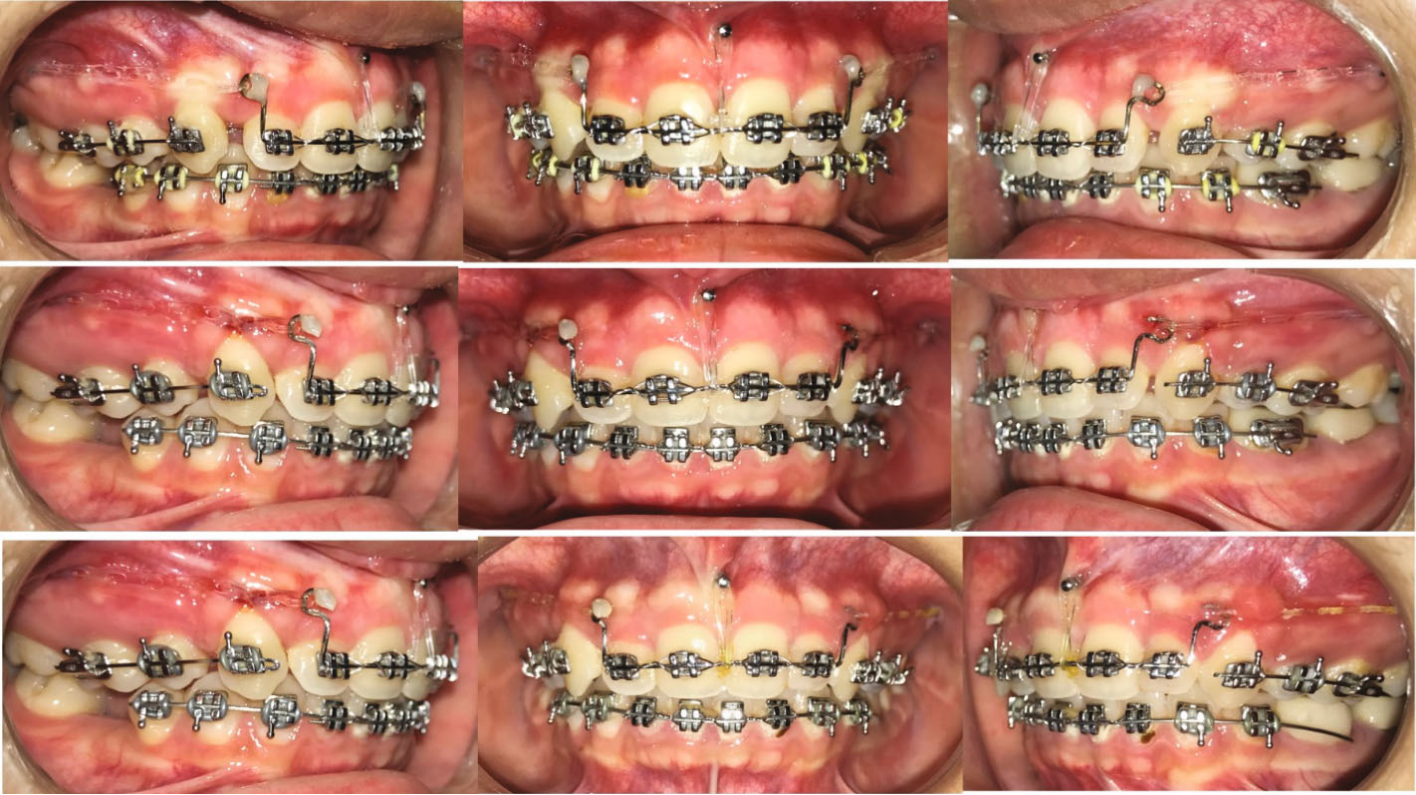
Retraction and simultaneous intrusion of anterior segment.

In six months, the anterior segment and the canines were retracted, and the deep bite was also corrected. The lower arch was stabilized passively with a 0.019^″^ × 0.025^″^ SS wire from the third month itself as the arch had leveled and the minor spaces had closed early in the treatment (Figure 4).

#### 2.3.3. Leveling and Alignment

**Figure 5:**
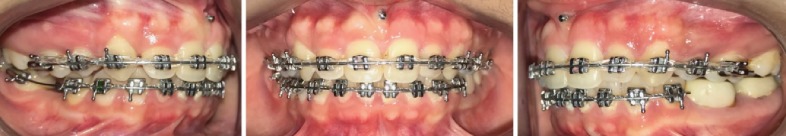
Finishing on a 0.019^″^ × 0.025^″^ SS archwire.

Following space closure, the arch was releveled using a 0.017^″^ × 0.025^″^ NiTi archwire for two months. The wire was tightly engaged in the bracket slots, and a power chain was placed from the upper first molar to the first molar on the other side to prevent space opening. This helped in paralleling the roots without opening up spaces (Figure 5).

#### 2.3.4. Finishing and Retention

**Figure 6:**
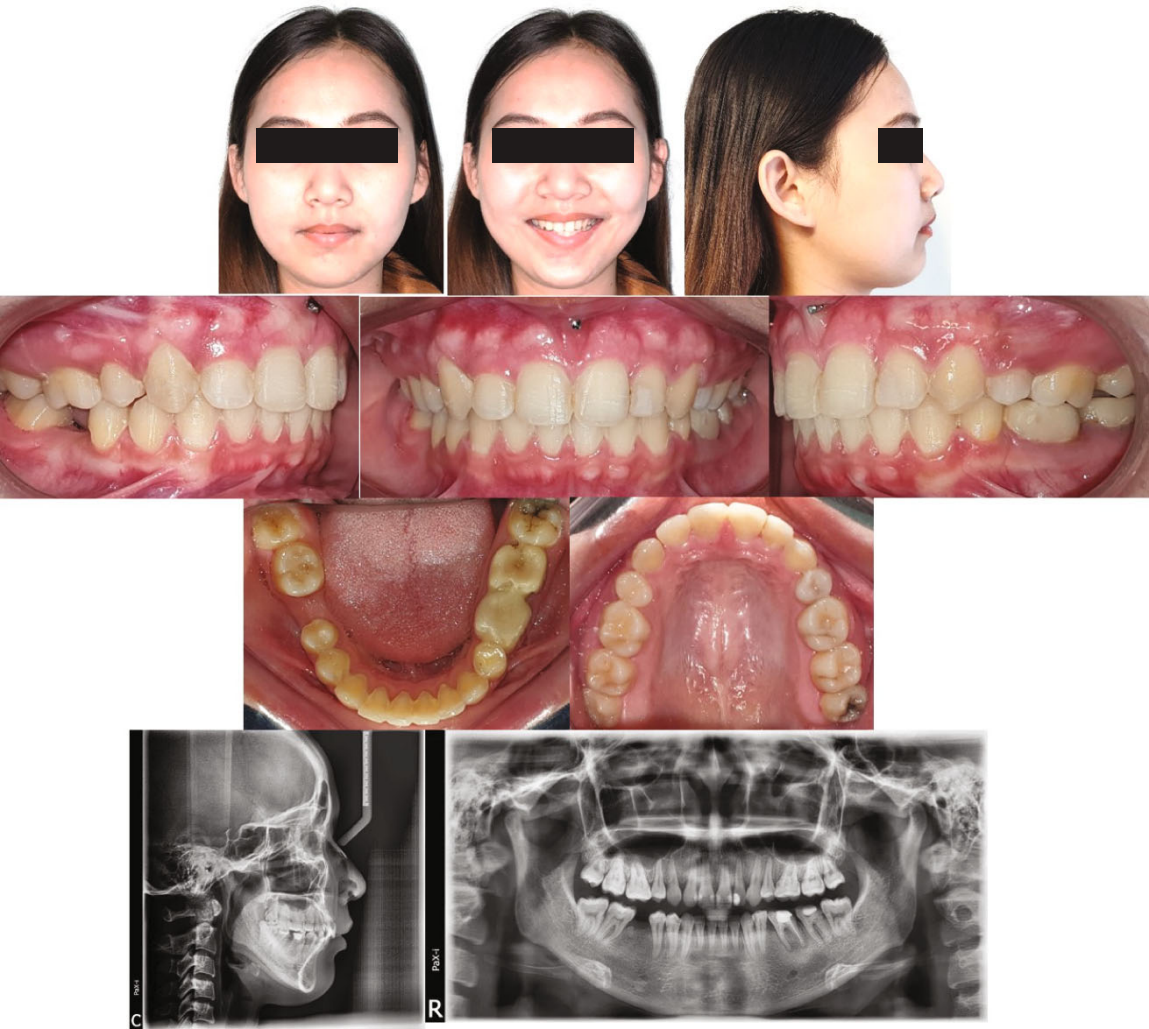
Posttreatment records.

Finishing was done on a 0.019^″^ × 0.025^″^ SS with light settling elastics following which the brackets were debonded. The anterior TAD was not removed and still kept in place to aid in the retention (Figure 6).

Essix retainers were planned on the upper and lower arches with the addition of a lingual button embedded in the upper Essix between the two central incisors. The patient was informed to wear the retainers for 24 hours for the next 2 years. In addition, she was instructed to attach a light intermaxillary elastic (3/16^″^, 2 Oz) from the anterior TAD to the button on the Essix at night for the next two years to retain the intrusion of the anterior segment.

## 3. Results

A balanced profile with an esthetic and pleasing smile was achieved along with harmony between the upper and lower lips, lip competence, and bilateral Class I canine relationships (Figure 6). The dental midlines were corrected, and no muscle or joint problems had developed during the treatment.

A panoramic radiograph taken after debonding showed acceptable root angulations with no evidence of root resorption, and stable bone levels.

**Figure 7:**
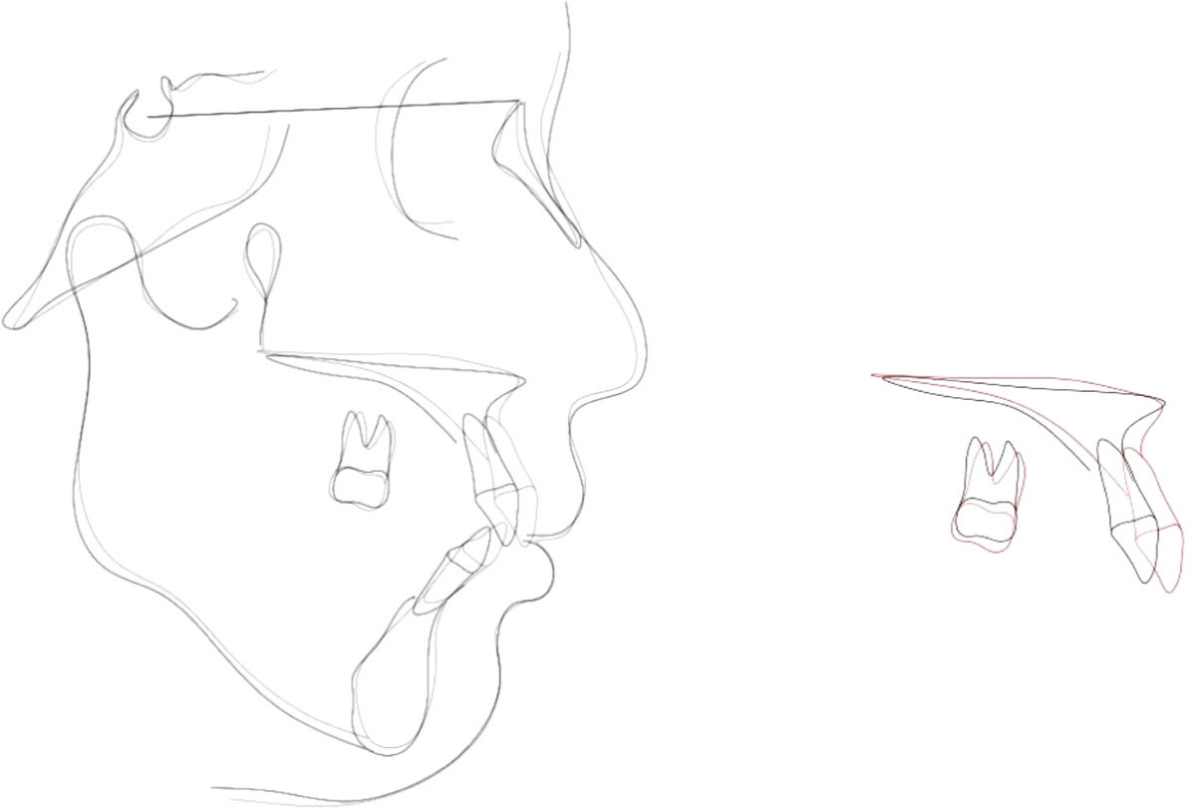
Superimpositions of pre- and posttreatment cephalograms showing almost constant mandibular planes, marked decrease in the upper and lower incisor inclination and upper incisor intrusion.

Posttreatment cephalometric analysis showed that the sagittal jaw relationship improved while facial height remained almost constant (Table 1). There was a marked decrease in the upper and lower incisor inclination (IMPA, 97.48°; U1-SN, 100.76°) (Figure 7). The overjet was corrected, addressing the patient's initial complaint of protrusive teeth and protruding lips.

## 4. Discussion

Poor choice of dental procedures, incorrect treatment indication, adoption of hazardous treatment strategies, inadequate treatment performance, wrong estimation of treatment time, not changing treatment plan when necessary, and not establishing good communication with the patient are failures that may significantly affect outcomes, quality, and stability of correction. Moreover, these factors delay the treatment by a significant amount of time [9].

Retracting teeth on a light, flexible, round NiTi wire with large forces causes a phenomenon called “roller coaster” effect [1]. The “roller coaster” effect is observed when a wire of low strength such as NiTi archwire is used for canine retraction. NiTi does not have the stiffness to remain rigid when a retracting force such as an elastic chain is stretched from the molar to the canine. The molar and premolar crowns tend to tip mesially and extrude distally. The flexible NiTi then bends gingivally and, as a result, tends to tip the canine crown distally. The orientation of the canine bracket when the crown tips distally tend to extrude the incisors and deepen the bite. The use of proper biomechanics to overcome the iatrogenic side effects and satisfactorily complete the case is paramount in such situations [10].

In our case, prior use of high forces to retract the upper canines bilaterally on a 0.016^″^ NiTi wire resulted in such “roller coaster” effects. In the recent decade, many studies have shown a surge in the usage of TADs as direct anchorage for correction of transmigrated lower canines [11], upper canine impactions [12], and molar uprighting [13] with the aid of sectional mechanics to nullify the unwanted side effects which accompany the use of archwire-guided mechanics or the usage of opposing dental components as anchorage.

The current clinical biomechanical setup would be more of a customization of the required mechanics to achieve the ideal treatment objectives (Figure 3). Since our main problem was time underscored by the demands and duration of preparing the anchorage, segregating the maxillary arch into 3 segments (1.6–1.3; 1.2–2.2; 2.3-2.6) was needed in order to prudently negate the demands of time, anchorage, and force vectors required to achieve the ideal treatment objectives. By performing this, the canines could be positioned in a Class I relationship by the independent utilization of the second premolars, and first molars reinforced with indirect anchorage from the posterior TADs as anchorage. The incisors could be retracted and intruded with the use of TADs, which could provide an ideal anchorage to support absolute anchorage demands and provide the ideal force vector required to correct the overjet and gingival show.

In the presented case, resistance to sliding, primarily binding of the wire in the bracket slot, was nullified with the use of loop and sectional mechanics. Power chains from the TADs posteriorly delivered forces of 150 grams to the anterior hooks per side, and the elastic thread from the third TAD (between the upper central incisors) delivered 60 grams of intrusive force to the anterior segment.

The advantage of this setup was that force delivered onto the anterior segment was completely utilized in the retraction and simultaneous intrusion unlike in archwire-guided mechanics wherein most of the force is unaccounted for and is probably lost in binding and notching [8].

Canine retraction was performed using a segment of 0.019^″^ × 0.025^″^ SS with closing loops distal to the canines with appropriate antirotational bends to prevent the distobuccal rotation of the canines.

Evidence has suggested that anterior intrusion is very hard to retain [14]. Therefore, it was decided to maintain the third TAD (between the upper central incisors) in place during the retention period, and the patient was instructed to wear 3/16^″^ 2 Oz elastics from the TAD to the lingual button incorporated in the Essix between the central incisors at night time during the retention phase.

In our case, this setup with sectional wires and TADs produced an uprighting, and intrusive effect on the upper incisors during space closure (9° reduction in the U1-SN). Additionally, the anchorage design avoided any significant change in the vertical dimension during sagittal correction of the Class II malocclusion (Figure 7 and Table 1). The occlusal plane remained almost stable (2° increase). There was a good amount of uprighting on the lower incisors (4° decrease in the IMPA) following lower space closure. The apt use of sound biomechanical principles helped us achieve all the treatment goals and objectives in under nine months.

## 5. Conclusion

We should always have in mind our limitations and the importance of proper orthodontic training while treating patients. This clinical biomechanical setup of sectional archwires with TADs helped us achieve all the treatment goals (canine retraction, retraction, and intrusion of the anterior segment) in a short period of time.

## Figures and Tables

**Table 1:** Cephalometric analysis.

Variable	Pretreatment	Posttreatment
FMA (dg)	28.94	29.43
FMIA (dg)	49.99	53.09
SNA (dg)	83.22	81.25
SNB (dg)	78.88	77.17
ANB (dg)	4.34	4.08
IMPA (dg)	101.07	97.48
Occlusal plane (dg)	5.54	7.30
U1-SN (dg)	109.45	100.76
Upper facial height	43.3%	42.6%
Lower facial height	57.7%	57.4%
